# Effect of a low-FODMAP diet for the management of functional abdominal pain disorders in children: a study protocol for a randomized controlled trial

**DOI:** 10.1186/s12937-020-00656-3

**Published:** 2021-01-02

**Authors:** Agata Stróżyk, Andrea Horvath, Jane Muir, Hania Szajewska

**Affiliations:** 1 Department of Paediatrics, The Medical University of Warsaw, Żwirki i Wigury 63A, 02-091 Warsaw, Poland; 2 Department of Gastroenterology, Monash University, Melbourne, Victoria Australia

**Keywords:** Child, Functional abdominal pain disorders, Irritable bowel syndrome, Diet, RCT

## Abstract

**Background:**

Evidence from studies in adults documents that fermentable oligosaccharides, disaccharides, monosaccharides, and polyols (FODMAPs) may be triggers of symptoms in individuals with functional abdominal pain disorders (FAPDs). However, in children, the evidence is very limited. We aim to assess the effects of a low-FODMAP diet compared with a regular diet for the management of children with FAPDs.

**Methods:**

We will perform a randomized, quadruple-blinded, controlled trial. Seventy-four children aged 8 to 18 years with a FAPD (Irritable Bowel Syndrome or Functional Abdominal Pain-Not Otherwise Specified), diagnosed according to the Rome IV criteria, will be randomly allocated to receive either a low-FODMAP diet or a regular diet for 4 weeks. The primary outcome will be the percentage of the responders, defined as the participants who have at least 30% improvement in abdominal pain intensity on a Visual Analogue Scale (VAS) during the last week of the trial compared with baseline, that is at least equal to the Reliable Change Index (≥ 25 mm change on VAS). Other outcomes will include changes in stool consistency, abdominal pain frequency, total scores on the Gastrointestinal Symptom Rating Scale, KIDSCREEN-10 Index and World Health Organization Five Well-Being Index, child’s school attendance and parents’ work absenteeism, and BMI-for-age z-score. Compliance, tolerability of the low-FODMAP diet, and adverse events also will be evaluated. Each FAPD subtype will be assessed separately.

**Discussion:**

There is a need for high-quality evidence regarding the dietary management of children with FAPDs. This randomized controlled trial (RCT) of rigorous methodological design will help to establish the effectiveness, if any, of a low-FODMAP diet for the management of FAPDs in the pediatric population. The findings of this RCT will assist with the development of guidelines and influence the direction of further research.

**Trial registration:**

NCT04528914

**Data and protocol version identifier:** 24/08/2020

**Supplementary Information:**

The online version contains supplementary material available at 10.1186/s12937-020-00656-3.

## Background

Functional abdominal pain disorders (FAPDs) affect 13.5% of children worldwide [1]. The Rome IV Criteria distinguished four types of FAPD: irritable bowel syndrome (IBS) (the most common, 8.8, 95% confidence interval [CI] 6.2–11.9) [1], abdominal migraine, functional dyspepsia, and functional abdominal pain – not otherwise specified (FAP-NOS) [2]. The main symptom of each is chronic abdominal pain which cannot be fully explained by any other medical condition. The diagnosis is symptom-based; however, it should be preceded by the exclusion of other diseases (e.g., celiac disease), especially if alarming symptoms occur.

The mechanisms underlying FAPDs are complex. Among them is a brain-gut axis disorder, resulting from the interaction of genetic, environmental, and psychological factors [2–4]. As the impact of FAPDs on school absenteeism, hospital admissions, psychosocial functioning of children, absenteeism of caregivers, and cost for families and healthcare providers is substantial [3–5], there is a need for evidence-based therapeutic options.

Whereas evidence from studies in adults suggests that fermentable oligosaccharides, disaccharides, monosaccharides, and polyols (FODMAPs) may be triggers of symptoms in individuals with FAPDs, evidence in children is very limited. However, a low-FODMAP diet is commonly prescribed for IBS in both adults and children, despite the limited evidence of its effectiveness [3]. The rationale for the aforementioned dietary restrictions is based on the assumption that a decrease in the short-chain fermentable carbohydrate load both prevents the osmotic effect of FODMAPs, resulting in a decrease in small intestine water volume, and limits the exaggerated fermentation of FODMAPs by colonic microbiota and associated gas production, which may alleviate the chronic abdominal pain [6, 7]. The FODMAPs are poorly absorbed in healthy people as well as in patients with FAPDs; however, a high intake of FODMAPs is associated with gastrointestinal symptoms only in the latter population, which is explained by the presence of visceral hypersensitivity and altered gut motility [8].

A 2017 updated Cochrane systematic review [3] found only one short-term (2 days), double-blind, cross-over, randomized controlled trial (RCT) investigating use of a low-FODMAP diet in children with IBS (*n* = 33) [9]. The authors reported fewer episodes of abdominal pain per day and no adverse events after 2 days of a low-FODMAP diet compared with both a traditional American childhood diet and the baseline value. Pain intensity improvement was reported for 8 responders and 10 placebo responders. For dietary modification of a single FODMAP, the evidence is also limited. A 2015 systematic review [10] found no effect of a lactose-free diet in children with recurrent abdominal pain (two RCTs). In both systematic reviews (Rutten [10] and Cochrane 2017 [3]), limited evidence was reported for a fructose-restricted diet (one RCT). A 2017 systematic review [11], which assessed the methodological quality of RCTs investigating use of a low-FODMAP diet in an age-mixed population with IBS, concluded that all included studies had domains with a high risk of bias. The short study duration, difficulties with blinding of study participants, and problems with selection of the control group were reported as the main study limitations. Further studies are needed, especially in children, which will address these limitations and determine the effectiveness of use of a low-FODMAP diet in children with FAPDs including IBS and FAP-NOS.

## Methods

This study was designed in accordance with Consolidated Standards of Reporting Trials (CONSORT) 2010 [12] and Standard Protocol Items: Recommendations for Interventional Trials (SPIRIT) 2013 [13] Statements (see Additional file 1). Additionally, the design of this study follows the guidelines of the Rome Foundation Working Group [14, 15], and pediatric subcommittee [16], as well as a core outcome set for clinical trials in pediatric FAPDs [17].

### Study objective and hypothesis

The primary aim of this RCT is to test the hypothesis that children with IBS and FAP-NOS who receive a low-FODMAP diet will have a lower mean abdominal pain intensity score compared with those who receive a regular diet after 4 weeks of intervention.

### Trial design

This study is a single-center, randomized, controlled, quadruple-blinded, superiority trial, with two parallel arms and allocation 1:1, as recommended [16].

### Participants, interventions, and outcomes

#### Study settings and recruitment

The recruitment of the study participants will take place among both inpatients and outpatients of the Department of Paediatrics of the Medical University of Warsaw, Poland (academic hospital). The start of the recruitment is planned in September 2020 and should be completed over 24 months. For study recruitment in a similar single-center study with similar eligibility criteria [9], 28 months was a sufficient period.

#### Eligibility criteria

##### Inclusion criteria

At enrollment, the volunteer children must fulfill all of the following eligibility criteria to be considered for inclusion: (1) age at least 8 years and ≤ 18 years (regardless of race, ethnicity, gender); (2) FAP-NOS or IBS diagnosed according to the Rome IV Criteria [2]; (3) baseline average pain intensity at least 30 mm on a 100-mm Visual Analogue Scale (VAS) [9]; (4) feeding via the oral route; (5) ability to read and comprehend any employed questionnaires/scales; (6) signed informed consent; and (7) stated availability throughout the study period.

Participants with IBS and a stool consistency score > 5 on the Bristol Stool Scale will be classified as having IBS with predominant diarrhea (IBS-D); if the stool consistency score is < 3, the subjects will be classified as having IBS with predominant constipation (IBS-C) [16].

##### Exclusion criteria

The exclusion criteria are as follows: receiving any other intervention/treatment with regard to FAP-NOS or IBS or those who received any other intervention during the last 3 months, an organic cause of symptoms or organic gastrointestinal disease, chronic illness, receiving medications which affect gastrointestinal motility, need for any other dietary management which could make the balancing or compliance with the diet troublesome, previously diagnosed carbohydrate intolerance without symptoms of FAPD after implementation of an exclusion diet, undernutrition (defined as World Health Organization [WHO] [18] growth charts < − 2 SD [standard deviation]), decreased growth velocity (sharp decline in growth line), or overweight or obese (> 1 or > 2 SD on the WHO growth charts, respectively), unintentional weight loss greater or equal to 5% of subject’ body weight (BW) within the previous 3 months [16], pregnancy, eating disorders, prior surgery of the gastrointestinal tract (within last 3 months), recurrent or unexplained fever, and developmental disabilities which impair the ability of the child to understand or communicate.

#### Intervention

**Fig. 1:**
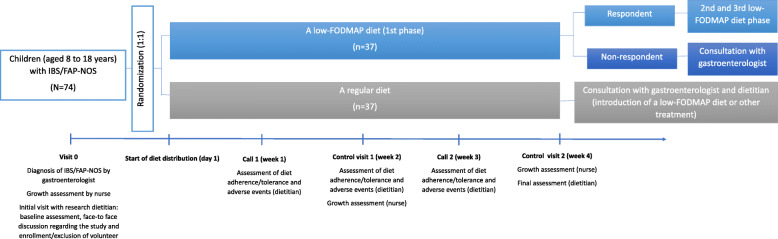
Flow chart of study design. Abbreviations: FAP-NOS, functional abdominal pain – not otherwise specified; FODMAPs, fermentable oligosaccharides, disaccharides, monosaccharides, and polyols; IBS, Irritable Bowel Syndrome; N, number of randomized participants; n, number of participants allocated to each group

All eligible children will be assigned to one of two groups following the randomization sequence. For the design of the intervention, please see Fig. 1. For 4 weeks, children will receive a low-FODMAP diet or comparator – a regular diet. This period is recommended for clinical practice [3, 19], as it is a sufficient period for the majority of patients to achieve symptom improvement and for the investigators to observe adverse events associated with intervention.

##### Diets

The low-FODMAP diet will contain the amount of FODMAPs that will not exceed the cut-offs for each FODMAP sugar per serving of food per sitting, as adopted from Varney et al. [20]. The regular diet will reflect the habitual FODMAP intake in a normal diet (following Krogsgaard et al. [11]). No changes, except for the modification of FODMAP intake in the intervention group, will be imposed; diets in both groups will be matched in terms of total energy, fat, protein, carbohydrates and dietary fiber with the usual participant’s diet. The requirements of daily energy and nutrients will be calculated as mean values based on a quantitative analysis of a 3-day food record to reflect the each child’s habitual diet. The nutritional value of each meal plan in both groups will be calculated, using dietary software (DIETA 6.0; http://www.izz.waw.pl [2018, Warsaw, Poland]). All meal plans will be checked for FODMAP content with the Monash FODMAP Calculator (www.monashfodmapcalculator.com.au/), including: excess fructose, lactose, sorbitol, mannitol, fructans, galactooligosaccharides, and oligosaccharides.

Participants will receive five meals each day (3 main meals: breakfast, dinner, and supper; and 2 additional smaller meals: lunch and dessert). All meal plans will be individually tailored by a dietitian after the initial assessment. In case of additional hunger, additional low-FODMAP snacks will be supplied with the meal delivery each Monday (i.e., portion of nuts, granola bars, gluten-free biscuits, or crackers).

Diets will be delivered by a catering company each morning to each child’s residence as an all-day box-diet. The information on the recommended storage and, if needed, preparation of each meal will be included.

##### Study procedure and timeline

For the study procedure and timeline, see Table 1 and Fig. 1. At the initial baseline visit (Visit 0), children will be diagnosed with IBS or FAP-NOS by a gastroenterologist, undergo a growth assessment by a nurse, and have an initial visit with a research dietitian. At the initial dietary consultation, all subjects will receive oral and written information regarding the study. A Monash FODMAP-trained dietitian will educate the caregiver and child about the principles of the study design, as well risks and benefits, during a face-to-face meeting. The subjects will be asked to complete a questionnaire, reflecting the preceding week, including questions about the intensity and frequency of abdominal pain, stool consistency, absence from school, and parents’ absence from work. Baseline scores on the Gastrointestinal Symptom Rating Scale (GSRS) [21], the KIDSCREEN-10 index [22] and the World Health Organization Five Well-Being Index (WHO-5) [18] will also be obtained. The child’s anthropometric measurements will be obtained by an independent nurse (see Growth in Outcome section). The baseline dietary assessment will include a qualitative and quantitative assessment of each child’s usual food intake based on a 3-day food record completed retrospectively, against the Nutrition Standards for the Polish population (2017) [23]. Based on the same food record, the baseline FODMAP intake in each meal also will be calculated using the Monash FODMAP Calculator. The dietitian will perform a thorough interview to tailor the diet to the individual child’s needs [19, 24, 25]. The baseline physical activity level also will be assessed using a Moderate-to-Vigorous Physical Activity (MVPA) screening measure [26].

**Table 1:**
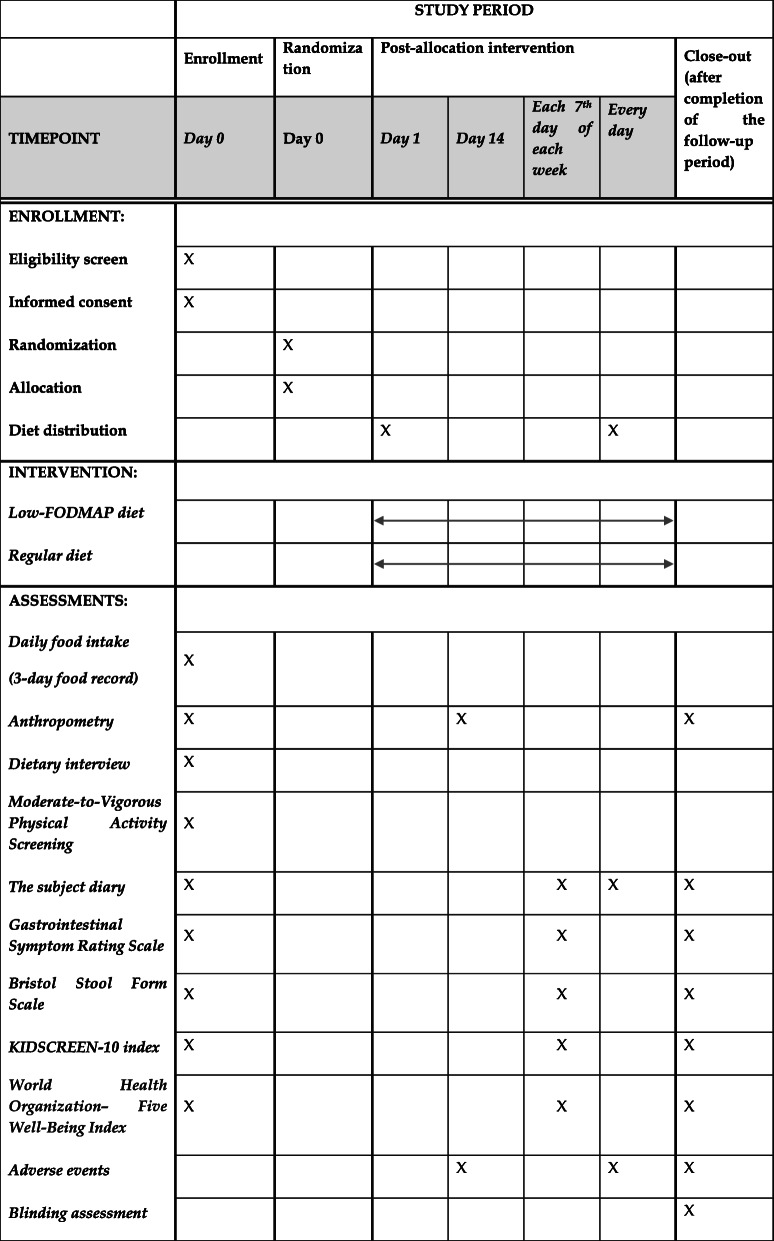
Participant timeline

Whenever a patient is enrolled, the participant’s case report form (CRF) will be created. Both, the CRF and one copy of the informed consent in paper form will be archived. Data from the CRF will be entered into an electronic database (see Data collection and management section).

Participant will be randomized and allocated to receive the intervention or control diet for 28 days. During the intervention period, the abdominal pain intensity (using the 100-mm VAS score), stool consistency (using the Bristol Stool Form Scale), pain frequency (by reporting the number of episodes/week), school attendance and parents’ work absenteeism (by reporting the number of days of school or work/week missed), and scores on the GSRS, KIDSCREEN-10 index and WHO-5 will be recorded in the subject’s diary each week (see Table 1). For children ≥14 years of age, the study diary will be filled out by themself; for younger children, the diary will be filled out by caregiver. Moreover, the child/caregiver(s) will be reporting the percent of each meal which the subject consumed (< 50%, 50–79% or 80–100%) each day, to estimate the amount of food consumed throughout the trial; the additional snacks consumed also will be reported daily (characteristics and amount). The tolerance of the diet will be assessed each day using a 100-mm VAS and reported in the subject diary. The caregiver and/or participants will be instructed to record in the study diary any observations concerning the health of the child, especially any symptoms which appeared close to a meal. The dietitian will also monitor the subjects’ acceptability of and adherence to the provided diet each week (see Fig. 1).

##### Concomitant care

Before enrollment, screening and baseline assessment of each subject is planned, as recommended [16]. All necessary laboratory, imaging, and endoscopic tests will be performed to rule out diseases that may mimic FAPD symptoms (e.g., celiac disease). Any psychological comorbidities also will be determined. In case of use of concurrent medication(s), the mechanism of drug action and potential influence on the intervention effect will be evaluated by a physician. However, enrolled subjects will be encouraged to maintain their usual dose and administration schedule of concomitant medications during the entire study period. No physical activity advice will be provided, as it is not the part of this intervention. During the study period, the caregiver(s) will be discouraged from starting any new dietary, pharmaceutical, or any other complementary or alternative treatments which could affect FAPD symptoms (e.g., selective serotonin reuptake inhibitors, unusual exercise, or probiotics). In case of any new treatment imposed, the participant or caregiver will be instructed to report this in the study diary. In case of the need for any rescue medication for FAPD, this will be prescribed by the investigator and noted in the CRF. Each commencement of new aforementioned therapy will be treated as a protocol violation and such patients will not be included in the per-protocol analysis.

##### Criteria for discontinuing the intervention

Withdrawal of informed consent is possible at any time, without any obligation for the child or caregiver to give reasons for the decision. Diet tolerance, compliance, and adverse events will be monitored during each control visit or call (see Fig. 1). Twenty-four phone contact to the study site will be available for all participants. An independent Data and Safety Monitoring Board (DSMB) will be appointed prior to study commencement to evaluate each reported adverse event (see Monitoring section). In case of occurrence of any serious adverse events or new circumstances affecting the safety of the child, the intervention will be discontinued. The reason and timing of any withdrawal (i.e., non-compliance, withdrawn consent, adverse events) will be documented.

##### Compliance

A controlled feeding trial, as is considered to be the gold standard for evaluating dietary interventions, may enhance the subjects’ adherence to the intervention and decrease confounding dietary habits. At the initial visit, each subsequent visit and during each call with the dietitian, participants will be encouraged to maintain adherence to the study protocol. Any individual issues associated with the diet will also be discussed. Compliance with the study protocol will be evaluated by aforementioned direct interview with child and/or caregiver and analysis of data from the subject diary. The percentage compliance in each group will be calculated by the dietitian. In agreement with other studies, a compliant participant is considered as one who consumes at least 80% of the provided diet [9, 15]. Snacks and meals outside the meal plan will be assessed separately.

##### Follow-up

All study outcomes (see below) will be assessed during the 4 weeks of the intervention. All subjects, after discontinuation or completion of the trial, will be encouraged to continue management of their FAP-NOS or IBS under the care of our department (if feasible, data from responders will be collected prospectively and analyzed, as a part of an independent study) (see Fig. 1).

#### Outcomes

In agreement with recommendations [16], one primary outcome was pre-specified:

- **Change in abdominal pain intensity.** Responders will be those with a daily reduction in the intensity of abdominal pain episodes of at least 30% at week 4 compared with baseline, and this value should be at least equal to the Reliable Change Index (RCI) for that sample [16, 27]. A cut-off of 25 mm for RCI change was adopted [16]. This outcome also will be assessed as the change at weeks 1, 2, and 3 from baseline.

For participant timeline, see Table 1.

Secondary outcomes to be assessed during the 4-week intervention, following the standardized core outcome set [17], will be:

- **Change in stool consistency.** Responders will be subjects with improvement in their average stool consistency during the last week of the trial compared with baseline [≥1 higher Bristol Stool Scale score in case of IBS-C, or at least one lower score in case of IBS-D] [16]. The change at weeks 1, 2, and 3 from baseline also will be measured.
- **Change in abdominal pain frequency** at weeks 1, 2, 3, and 4 from baseline [16].
- **Change in GSRS** [21] **total score** at weeks 1, 2, 3, and 4 from baseline.
- **Change in KIDSCREEN-10 index** [22] **total score** at weeks 1, 2, 3, and 4 from baseline.
- **Change in WHO-5** [18] **total score** at weeks 1, 2, 3, and 4 from baseline.
- **Change in percentage of school attendance** associated with IBS symptoms (at weeks 1, 2, 3, and 4 from baseline).
- **Change in percentage of parents’ work absenteeism** associated with IBS symptoms in child (at weeks 1, 2, 3, and 4 from baseline).
- **Change in BMI-for-age z-score**. The BW (kg) and standing height (cm) will be measured following standard methods by an independent nurse at baseline, after 2 weeks, and after 4 weeks. Body Mass Index (BMI) will be calculated using the standard eq. BMI-for-age z-score will be computed using the WHO AnthroPlus software v1.0.4., then assessed and monitored over time using the WHO growth charts by a dietitian.
- **Percentage of compliant participants** (see Compliance section).
- **Percentage of tolerability of the low-FODMAP diet.** This outcome will be reported as a mean for each study group, and as a comparison between groups (MD [mean difference] with 95% CI).
- **Adverse events.** The number of all adverse events and the number of participants reporting adverse events associated with the intervention (with characteristics) will be reported for each study group (see Criteria for discontinuing the intervention and Harms sections).

#### Sample size

The primary outcome is the reduction in the abdominal pain intensity in the low-FODMAP diet group compared with the regular diet group over the 4-week intervention. We assumed a 30% difference as being a clinically significant primary endpoint [16]. We estimate that 31 children in each group are needed to achieve a significant difference with a power of 80%, along with a two-sided 5% significance level. Given the anticipated dropout rate of 20%, we estimate 37 subjects are necessary in each study arm. Sample size calculations were performed with StatsDirect (StatsDirect Ltd. StatsDirect statistical software. http://www.statsdirect.com. England: StatsDirect Ltd. 2013).

### Assignment of interventions

#### Sequence generation and allocation concealment

Randomization will be based on a computer-generated randomization list developed by an independent investigator with no clinical involvement in the trial. The random numbers will be generated in blocked randomization (using a random block of 4: 2 subjects in group A and 2 in group B) with a 1:1 allocation ratio to ensure good balance between participant characteristics in both groups. The randomization sequence will be created using StatsDirect statistical software. Stratification will not be performed.

Allocation concealment will be ensured using opaque, sealed, numbered envelopes. The data regarding intervention assignments will be stored in a sealed envelope in a safe in the administrative part of the department. An independent person will open the next consecutively number envelope. Information about allocation of each participant will be given to the dietitian. The dietitian will develop a meal plan for each enrolled participant following the allocation. Each meal plan will contain the full name and address of the child. The meal plans will be provided to the catering company. The catering company will not be informed about the type of diet, and no suggestive information will be attached to the all-day box-diet provided to the participant.

After performing the data analysis, code numbers will be opened by the research coordinator (AH). In case any medical problem occurs for which treatment allocation is needed, the code can be broken by a principal researcher (AH, HS).

#### Blinding

This is a quadruple-blinded trial. In addition to the subjects, the research investigators (AH, HS), outcome assessor (AH), and independent statistician (data analyst) will be blinded until the completion of the data analysis. The participants and their caregivers will be not informed about the characteristics of the diet being consumed (the name and description will not be provided, as in similar previous RCTs [28, 29]). The researcher will encourage the caregivers/participants not to attempt to identify the nature of the provided diet using available data sources; however, an associated bias cannot be excluded. Therefore, after completion of the follow-up period, each participant and outcome assessor will be asked whether he/she believes that active treatment was administered (these data will be published with the final results, as recommended) [15]. The dietitian (AS) will remain unblinded. The procedure was established to maintain separation between the dietitian and outcome assessor and analysts. The primary outcome will be assessed by a blinded researcher (AH). If possible, to increase the similarity of diets in the two groups, low-FODMAP substitutes will be provided in place of high-FODMAP products in the meal plans.

### Data collection, management, and analysis

#### Data collection and management

Each study participant will be assigned a study identification number. A CRF, containing the participant’s identification number and necessary baseline data, will be completed in paper form. All data will be checked for completeness, entered, and stored in a password-protected electronic database. All original copies of CRFs and all study data in paper form will be stored in a locker within the study site, available for the involved researchers only. Each CRF must be completed, checked for completeness of data, and signed by a researcher. Only the research team and person responsible for statistical analysis of the data will have access to the full trial dataset (blinded of any identifying participant).

#### Data analysis

All analyses will be conducted on an intention-to-treat basis, including all patients in the groups to which they are randomized for whom outcomes are available (including dropout and withdrawals). Per-protocol analysis also will be performed, in which all subjects who finish the study according to the protocol will be included.

Descriptive statistics will be used to summarize baseline characteristics. The Student t-test will be used to compare mean values of continuous variables approximating a normal distribution. For non-normally distributed variables, the Mann-Whitney U test will be used. The x^2^ test or Fisher exact test will be used, as appropriate, to compare percentages. The same computer software will be used to calculate the relative risk (RR), number needed to treat (NNT), and mean difference (MD), all with a 95% confidence interval (CI). The difference between study groups will be considered significant when the *p* value is < 0.05, when the 95% CI for RR does not include 1.0, or when 95% CI for MD does not include 0. All statistical tests will be two-tailed and performed at the 5% level of significance.

#### Methods of additional analyses

Both the primary and secondary outcomes will be analyzed by logistic regression, controlling for three, prespecified, potential confounding variables, i.e., age, sex, and subtype of IBS. The difference between study groups will be considered significant when the *p* value is < 0.05, when the 95% CI for RR (or odds ratio [OR]) does not include 1.0, or when 95% CI for MD does not include 0. All statistical tests will be two-tailed and performed at the 5% level of significance.

#### Missing data

Every effort will be made to minimize missing baseline and outcome data. However, the amount of missing data will be reported for each study arm. The reasons for withdrawals, if feasible, will be compared qualitatively between groups. Multiple imputation methods for missing data will be included to address any missing data, as recommended [13, 30, 31].

### Monitoring

#### Data and participant monitoring

The study will be performed following the protocol registered before the commencement of screening. However, in case of unexpected and valid circumstances, the protocol registry will be immediately updated and, if required, the bioethical committee will be informed.

The independent DSMB will review data after recruitment of 25, 50, and 75% of participants to review the study progress (including rate of recruitment and completeness of data) and all reported adverse events. The number of recruited children will be monitored and kept up to date. In case of an insufficient recruitment rate, defined as associated with the risk of not finishing the study within the established time (which is 2 years), we will advertise our trial among other pediatric wards and primary care physicians, however, only if adequately trained personnel is present.

#### Harms

Although the intervention is expected to be safe, all adverse events will be reported in the CRF, classified (following the International Conference on Harmonisation *Good Clinical Practice* guidelines), and evaluated for duration, intensity, and causal relationship with the intervention. Each serious adverse events will be reported to the project leader within 24 h of the notification. The project leader will notify, within 7 days, the bioethical committee, all investigators involved in the study, and the catering company responsible for the diet provision. Due to the risk of potential nutritional imbalance and growth impairment, the intervention will be guided by professionals with expertise in dietary management.

Due to the study design, involving the delivery of study diets by an outside company to each participant’s residence, the appropriate written permission of each caregiver will be obtained. Each caregiver will receive oral and written information with regard the collection and processing of personal data during and after their child’s participation in this trial.

### Ethics and dissemination

#### Research ethics approval

This protocol and the informed consent form was approved by the Ethics Committee of the Medical University of Warsaw.

#### Protocol amendments

Any modification to this version of protocol which may affect study design, sample size, study procedures, as well as benefits and harms for the participant will require a formal amendment to the protocol. Each amendment must be approved by the Ethics Committee of the Medical University of Warsaw prior to implementation.

#### Consent or assent

Written informed consent will be obtained from all participants before enrollment. The research dietitian will have a discussion with child and the child’s caregiver and answer any questions regarding the study. For children ≥13 years of age, the participants themselves can sign the informed consent; for younger children, two copies of the written informed consent will be obtained from the child’s caregiver.

#### Confidentiality

Each participant will have a participant’s identification number to maintain confidentiality. The electronic database and any electronic records will be password-protected and/or kept in password-protected folders (see Data collection and management section).

#### Access to data

Access to paper and electronic study records will be limited only to the researchers involved in this trial (AS, AH). An external independent statistician will receive an access to de-identified electronic dataset.

#### Ancillary and post-trial care

The compensation of harms will be covered by study insurance policies. For post-trial care, see Follow-up section and Fig. 1.

#### Dissemination policy – trial results

The findings of this RCT will be submitted to a peer-reviewed journal, no later than a year after the post-intervention data collection. The abstract will be submitted to relevant national and international conferences. No later than 3 years after data analysis, a full, anonymized, participant-level data set will be archived for sharing purposes. Data analysis will be completed over 3 months after RCT completion.

## Discussion

Currently, there are no evidence-based guidelines on the diagnosis and management of FAPDs in children. According to evidence in adults, use of a low-FODMAP diet is promising, however, in children the evidence is scarce [3]. RCTs involving a low-FODMAP diet are also affected by several methodological limitations (e.g., choice of control group) [11].

This study will assess the effects of a low-FODMAP diet compared with a regular diet for the management of children with FAPDs (IBS, FAP-NOS). Although high-quality evidence is needed, no study is perfect, as every RCT investigating dietary modifications of FODMAPs has had some limitations. The risk of bias associated with the possibility of the participant or caregiver guessing the nature of the provided diet using other sources cannot be excluded. To minimize this bias, a post-intervention assessment of blinding success, of both participants and researchers, has been planned (see Blinding section). A second limitation is this study involves evaluation of the first phase of a low-FODMAP diet only, without long-term follow-up. Thus far, no study conducted in children has assessed all three phases of administering a low-FODMAP diet in children with FAPDs. However, based on the clinical and research experience of this research center, such a long-term study is judged to be difficult to conduct in the pediatric population. Therefore, we decided to investigate the effectiveness and safety of only the first phase of use of a low-FODMAP diet in this RCT; however, all participants will be encouraged to continue the management of their FAPDs under the care of our research team (see Follow-up section). If feasible, clinical data from participants who choose to continue the low-FODMAP diet at our center will be collected prospectively as an independent study.

This RCT of rigorous methodological design will help to establish the effectiveness, if any exists, of the use of a low-FODMAP diet in the management of FAPDs in the pediatric population. This study will be conducted by a research center with experience in conducting both independent RCTs and trials in children with FAPDs, and in cooperation with a person who is experienced in research involving FODMAPs. The findings of this RCT will fill a gap in current evidence and inform guideline development groups about the actual efficacy of a low-FODMAP diet in the management of FAPDs in children. This RCT may also guide the direction of further research in this area.

## Supplementary Information

**Additional file 1.** SPIRIT 2013 Checklist.

## Data Availability

The datasets used and/or generated during this study will be made available from a given author upon reasonable request, after the publication of results, no later than 3 years from the completion of data analysis.

## Abbreviations

BMI: Body Mass Index
BW: Body weight
CI: Confidence interval
CONSORT: Consolidated Standards of Reporting Trials
CRF: Case report form
DSMB: Data and Safety Monitoring Board
FAPDs: Functional abdominal pain disorders
FAP-NOS: Functional abdominal pain – not otherwise specified
FODMAPs: Fermentable oligosaccharides, disaccharides, monosaccharides, and polyols
GSRS: Gastrointestinal Symptom Rating Scale
IBS: Irritable bowel syndrome
IBS-C: IBS with predominant constipation
IBS-D: IBS with predominant diarrhea
MD: Mean difference
MVPA: Moderate-to-Vigorous Physical Activity
OR: Odds ratio
RCI: Reliable Change Index
RCT: Randomized controlled trial
RR: Relative risk
SD: Standard deviation
SPIRIT: Standard Protocol Items: Recommendations for Interventional Trials
VAS: Visual Analogue Scale
WHO: World Health Organization
WHO-5: World Health Organization Five Well-Being Index
